# DNA Binding Properties of the Small Cascade Subunit Csa5

**DOI:** 10.1371/journal.pone.0105716

**Published:** 2014-08-22

**Authors:** Michael Daume, André Plagens, Lennart Randau

**Affiliations:** Prokaryotic Small RNA Biology, Max Planck Institute for Terrestrial Microbiology, Marburg, Germany; Keio University, Japan

## Abstract

CRISPR-Cas systems provide immunity against viral attacks in archaeal and bacterial cells. Type I systems employ a Cas protein complex termed Cascade, which utilizes small CRISPR RNAs to detect and degrade the exogenic DNA. A small sequence motif, the PAM, marks the foreign substrates. Previously, a recombinant type I-A Cascade complex from the archaeon *Thermoproteus tenax* was shown to target and degrade DNA *in vitro,* dependent on a native PAM sequence. Here, we present the biochemical analysis of the small subunit, Csa5, of this Cascade complex. *T. tenax* Csa5 preferentially bound ssDNA and mutants that showed decreased ssDNA-binding and reduced Cascade-mediated DNA cleavage were identified. Csa5 oligomerization prevented DNA binding. Specific recognition of the PAM sequence was not observed. Phylogenetic analyses identified Csa5 as a universal member of type I-A systems and revealed three distinct groups. A potential role of Csa5 in R-loop stabilization is discussed.

## Introduction

CRISPR RNAs (crRNAs) are the key elements of a prokaryotic immune system in defending against invading mobile genetic elements termed CRISPR (clustered regularly interspaced short palindromic repeats)-Cas (CRISPR-associated genes) [1]. This CRISPR-Cas system is the only adaptive immune system in prokaryotes known so far. Its defense response acts specifically on DNA or RNA sequences originating from previously encountered invaders, while other known innate prokaryotic anti-invader systems, e.g. the R-M (restriction-modification) or Abi (abortive infection) systems, act non-specifically [2].

The type I CRISPR-Cas mechanism is divided into three steps - acquisition, expression and interference. During acquisition, a protein complex containing Cas1 and 2 binds the invading nucleic acid, e.g. phage DNA, and recognizes a sequence motif consisting of few nucleotides, named the protospacer-adjacent motif (or PAM-motif) [3]–[6]. In a subsequent processing step, a sequence of defined length adjacent to the PAM, called the protospacer, is predicted to be excised and incorporated into an expanded CRISPR array as a new spacer [2], [7]–[9]. In the expression stage of the CRISPR-Cas mechanism, the CRISPR array is transcribed into a long precursor RNA, the pre-crRNA, which is then processed into short, mature crRNA by Cas6 [1], [10]–[12]. Finally, during the interference stage, a complex of several Cas proteins binds the crRNAs [1], [10], [13]. The complementarity of the spacer sequence of the crRNA to an invasive sequence, during a repeated encounter, guides this interference complex to the target site [14], [15]. Once bound, the associated Cas3 protein cleaves the targeted sequence, leading to its degradation [13], [14], [16]–[19].

Computational analysis of *cas* gene families defined three basic CRISPR-Cas types (type I, type II, type III), which are further divided into at least eleven subtypes (type I-A to F, type II-A to C, type III-A and B) [20], [21]. While all three main types encode the conserved *cas1* and *cas2* genes involved in acquisition, they most notably differ in the machinery responsible for pre-crRNA processing and interference. Type I CRISPR-Cas systems are defined by the signature protein Cas3, comprising a histidine/aspartate (HD)-nuclease domain and a DExH helicase domain [18], [20], and the crRNA-guided multi-protein complex called Cascade (CRISPR-associated complex for antiviral defense) [1], [22]. During interference, this complex drives the formation of the R-loop structure in the bound, double-stranded DNA (dsDNA) via complementary base pairing of the crRNA with the target DNA strand [22]. The DNA is then unwound and cleaved by the recruited Cas3 [14], [18], [19], [23].

The genome of *T. tenax* encodes 23 conserved *cas* genes adjacent to seven CRISPR loci, classified as two type I-A and one type III-A CRISPR-Cas systems [24]. The previously analyzed I-A Cascade of *T. tenax* is encoded by an operon (TTX_1250–1255) consisting of the subtype-specific *cas* genes *csa5* and *cas8a2*, and the core *cas* genes *cas7*, *cas5a*, *cas3*′ *and cas3″* [24]. While structural features and the interference mechanism of the type I-E Cascade of *Escherichia coli* have been studied extensively, much less is known about type I-A Cascade activity. Recently, we established an *in vitro* assembly of the I-A Cascade from six recombinant Cas proteins, synthetic crRNAs and target DNA fragments [25]. The assembly of the type I-A Cascade indicated that the split Cas3 domains Cas3′ (helicase) and Cas3″ (DNA nuclease) are an integral part of this complex [25].

During the interference reaction, self- and non-self discrimination is crucial to ensure degradation of the exogenous DNA. Thus, scanning for the PAM on a dsDNA target by Cascade is thought to be the initial step during CRISPR-Cas interference [26], [27]. In the type I-E Cascade, the L1 loop domain of Cse1 (CasA) was shown to be required for non-self target recognition by interacting with the PAM, and was found to be essential for the Cas3-mediated degradation [26], [28], [29]. For the type I-A CRISPR-Cas system, a PAM recognition protein could not yet be identified. The functional role of the two Cascade proteins Csa5 and Cas8a2, in analogy to type I-E often referred to as small and large subunits, respectively, remains elusive, but both proteins are proposed to bind DNA [30]. The crystal structure of *Sulfolobus solfataricus* Csa5 exhibits an α-helical domain that shows homology to the C-terminal domain of the small subunit Cse2 (CasB) from the type I-E systems of *Thermus thermophilus* and *Thermobifida fusca* [31]. Cse2 forms a dimer in Cascade, which is hypothesized to stabilize the R-loop structure by binding either the RNA:DNA heteroduplex or the displaced strand [22], [32]. However, *S. solfataricus* Csa5 was not observed to interact with nucleic acids; rather, the protein was suggested to play a different role in Cascade, in contrast to Cse2 [31]. Furthermore, the Csa5 crystals exhibited a striking oligomerization pattern that involved the formation of salt bridges [31].

Here, we present the biochemical characterization of the *T. tenax* Cas protein Csa5. We identified Csa5 as the small Cascade subunit present as a universal member of type I-A systems. Csa5 was shown to bind nucleic acids with a preference for single-stranded DNA, and we identified conserved residues involved in this interaction. Sequence-specific binding was not observed. Csa5 formed oligomers that abolished DNA binding. We hypothesize that Csa5 might play a role in R-loop stabilization, which would coincide with its exclusive presence in thermo- and hyperthermophilic Archaea.

## Materials and Methods

### Phylogenetic analyses

159 archaeal genomes represented in the CRISPI database were analyzed for the presence of a subtype I-A CRISPR-Cas region [33]. The I-A *cascade* operons were identified in 46 genomes, characterized by the gene order *cas7*, *cas5*, *cas3*′, *cas3″* and *cas8a* (or annotated as: *csax*). In all genomes the gene sequence adjacent to *cas7* within the operon structure was defined as a *csa5* candidate and further subjected to phylogenetic analyses (in total 54 sequences). Phylogenetic analysis was carried out with the phylogeny.fr web server [34], by multiple sequence alignments (MUSCLE), alignment curation including manual adjustments, construction of the phylogeny (PhyML) and visualisation of the tree (DrawTree). For simple homology searches of DNA and protein sequences the BLAST tool was used [35]. The prediction of the Csa5 structure was performed with the I-TASSER platform [36].

### Cloning, mutagenesis and production of Csa5

The *T. tenax* Kra1 *csa5* wild-type gene construct (TTX_1250) was available in the vector pET24a (Novagen) backbone [24]. The *csa5* mutants were generated by site-directed mutagenesis using the QuikChange protocol according to the instructions of the manufacturer (Stratagene). Oligonucleotides for mutagenesis were designed with Agilent's Primer Design Tool (Table S1). Mutations were verified by sequencing (MWG Eurofins). All generated pET24a+*csa5* vectors were transformed into *E. coli* Rosetta(DE3)pLysS cells for recombinant protein production. Cultivation of *E. coli* was carried out in Erlenmeyer flasks by shaking (200 rpm) at 37°C in lysogeny broth (LB) medium containing the appropriate antibiotics. Protein production was induced by the addition of 1 mM isopropyl β-_D_-1-thiogalactopyranoside (IPTG) after growing the cells to an OD_600_ of 0.6–0.8.

### Purification of Csa5

The recombinant Csa5 protein was purified as described before [25]. Briefly, *E. coli* cell pellets were lysed in purification buffer (100 mM HEPES/KOH pH 7, 10% glycerol, 10 mM β-Me (β-mercaptoethanol), 300 mM NaCl), heat precipitated (30 min, 90°C), purified via affinity chromatography using a Blue-Sepharose column (HiScreen Blue FF, GE Healthcare) and via anion-exchange chromatography using a MonoQ column (MonoQ 5/50 GL, GE Healthcare) on a FPLC system (ÄKTApurifier, GE Healthcare). The protein concentration was analyzed by the Bradford assay (BioRad). The purity of the elution fractions was analyzed by sodium dodecyl sulfate–polyacrylamide gel electrophoresis (SDS–PAGE) using 15% SDS-gels and Coomassie Blue staining.

### Size determination of the recombinant Csa5 variants

The determination of the native molecular weight of monomeric and oligomerized protein was carried out by size exclusion chromatography of 1–3.25 mg protein using a gel filtration column (Superdex 200 10/300GL, GE Healthcare), which was equilibrated in purification buffer. The molecular size of the proteins was determined with the help of calibration proteins (Gel Filtration Markers Kit for Protein Molecular Weights 12–200 kDa, Sigma-Aldrich).

### Electrophoretic mobility shift assay (EMSA)

The DNA- and RNA-binding activity of Csa5 was studied by electrophoretic mobility shift assays. Radiolabeled ssDNA oligonucleotides and *in vitro* transcribed mature crRNA were used as substrates. The ssDNA oligonucleotides (Table S2) were ordered from MWG Eurofins. Generation of the synthetic mature crRNA (Table S2) was performed as described before [25]. The substrates were labeled in a T4-PNK reaction using [γ^32^P]-ATP (5000 ci/mmol, Hartmann Analytic). For the generation of dsDNA, complementary labeled and cold ssDNA oligonucleotides were hybridized in a mixture of 1∶1.5 in water at 95°C for 5 min and then slowly cooled down to RT. 15,000 cpm of labeled substrate and 3.75 to 60 µM Csa5 were mixed in a volume of 10 µl EMSA binding buffer (100 mM HEPES/KOH pH 7, 10 mM β-Me). The reactions were incubated for 30 min at 37°C and then separated on a 6% non-denaturing TBE polyacrylamide gel. The detection of radioactivity was carried out by phosphorimaging. Gel band analysis was performed with ImageQuant 5.2 software.

### Microscale Thermophoresis

The dissociation constant K_d_ of the binding of Csa5 WT to non-target ssDNA was determined by microscale thermophoresis (MST). Purified Csa5 WT was dialyzed overnight in MST optimized buffer (50 mM Tris-HCl pH 7.4, 150 mM NaCl, 0.05% Tween-20) and adjusted to a concentration of 160 µM (2.41 mg/ml). A serial dilution of 3∶4 of the protein was performed in 16 reaction tubes with a final volume of 10 µl. Ten µl of Cy3-labeled non-target ssDNA were added to the reactions in a final concentration of 10 nM, resulting in a protein concentration of 60 µM for the first reaction tube. The reactions were incubated for 30 min at 37°C. Afterwards, the reactions were filled in standard type glass capillaries (Nanotemper) and analyzed in the MST instrument (Monolith NT.115, Nanotemper) using the following settings: LED power 95%, MST Power 80%. The reactions were measured in triplicate.

### Deoligomerization approaches

Deoligomerization of a nine month-old, highly multimerized Csa5 solution was attempted by GdmCl- (guanidinium chloride) and temperature-treatment. An aliquot of 500 µl protein solution was adjusted to a concentration of 6 M GdmCl. One half of the sample was incubated for 30 min at RT, the other one at 95°C. Subsequently, the samples were dialyzed overnight in purification buffer. Another aliquot of 500 µl protein solution was mixed with SDS-sample buffer and incubated for 5 min at 120°C in an autoclave. The samples were analyzed by SDS-PAGE and Coomassie staining.

### Reconstitution and *in vitro* Cascade interference assays

The reconstitution of the mature Cascade complex was performed as described previously [25]. Equal amounts (300 µg) of each GdmCl-solubilized protein Cas5a, Cas3′, Cas3″ and Cas8a2 were mixed with the purified proteins Cas7 and either the Csa5 wildtype or the Csa5 mutants (Y29A, D33A and D30A/D33A) and co-refolded via stepwise dialysis into GdmCl-free buffer. Aggregated proteins were precipitated (14,000×g, 30 min, 4°C), soluble proteins concentrated with centrifugal filter units (MWCO: 10 kDa), protein concentration measured and further analyzed by SDS-PAGE. For interference tests, 500 nM refolded Cascade were loaded with 500 nM crRNA (crRNA 5.2, Table S2) and the cleavage reaction started by the addition of 2 nM crRNA-matching 5′-labeled ([γ-^32^P]-ATP (5000 ci/mmol, Hartmann Analytic)) hybridized dsDNA (for/rev: int 5.2_CCT, Table S2), as described previously [25]. The reaction products were separated on 20% denaturing TBE-polyacrylamide gels alongside the low molecular weight marker (10-100 nt, Affymetrix).

## Results and Discussion

### Computational identification and phylogeny of the small Cascade subunit Csa5

**Figure 1:**
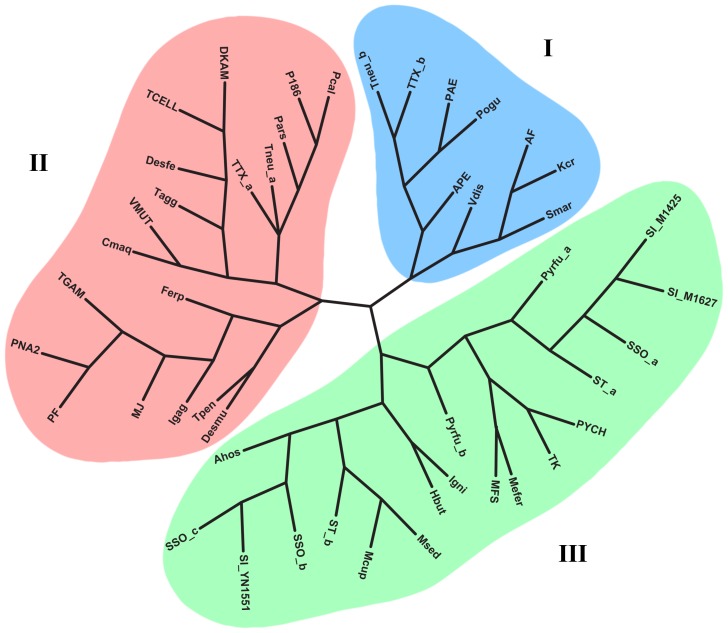
Phylogenetic tree of Csa5. Phylogenetic analyses of all identified Csa5 candidates revealed a clustering of the sequences into three distinct groups (group I–III). Depicted are the genome tags of the organisms (for details see Table S3). Multiple copies of Csa5 candidates are marked with a-c according to their position in the genome.

BLAST searches of the *T. tenax* Csa5 (TTX_1250) sequence only identified homologs of close relatives of the genus *Pyrobaculum*, while a significant similarity to the characterized *S. solfataricus* Csa5 protein (SSO1398) was not detectable. Additionally, structural analysis with the I-TASSER server could not identify reliable protein structures that showed homology to the Csa5 of *T. tenax* [36]. Due to this low conservation and the striking diversity of Csa5 sequences, we strived to obtain a broader overview of the distribution of *csa5* genes in archaeal subtype I-A CRISPR-Cas systems. Therefore, we analyzed the genomic context of 159 archaeal genomes and could identify subtype I-A *cascade* operons in 46 genomes, characterized by the split of Cas3 into the two subunits *cas3*′ and *cas3″* and the presence of the marker gene *cas8a*. Notably, this I-A CRISPR-Cas subtype was only identified in thermo- and hyperthermophilic Archaea. In every genome, adjacent to Cas7, a small protein with a length of 86–169 amino acids, often annotated as hypothetical, was identified (in total 54 times with up to three copies per genome; see Table S3). In addition, a previously unknown second homolog of *T. tenax* Csa5 (TTX_0236) could be identified, located within a second conserved I-A *cascade* operon. In the following, we define the two Csa5 homologs due to their position in the genome as Csa5a (TTX_0236) and Csa5b (TTX_1250). A multiple sequence alignment (MUSCLE) of all Csa5 candidates revealed the previously noted conserved amino acids D (Csa5b: position D33) and R (Csa5b: position R51) to be present in nearly all sequences (Fig. S1) [31]. A phylogenetic analysis of these sequences using PhyML revealed branching into three main Csa5 groups (Fig. 1). We identified several amino acid residues that exhibit different degrees of conservation for these three Csa5 protein groups. A conserved Y residue (Csa5b: position Y29) can be identified in groups I and II, but is mostly missing in group III. A conserved L residue (Csa5a: position L19) is found in groups II and III, but is absent in members of group I. At the C-terminus of the Csa5 sequences, an Alanine-rich region can be observed. The *T. tenax* Csa5b clustered with its near-homolog of *Pyrobaculum neutrophilum* (Tneu_1137) into group I. Csa5a of *T. tenax* is located in group II, again clustering with the second homolog of *P. neutrophilum* (Tneu_0993). In group III, the previously analyzed Csa5 of *S. solfataricus* (SSO1398) is located together with other Csa5 members from *Sulfolobales*. By analyzing the genomic context of the archaeal subtype I-A CRISPR-Cas systems, Csa5 could be identified in every *cascade* operon, which indicates a conserved function of this subunit within the interference complex. Even though the sequences show exceptional divergence, three main subfamilies of Csa5 were identified, which should help to identify other *csa5* genes encoding a universal small Cascade subunit.

### Csa5 shows preferred binding to ssDNA

**Figure 2:**
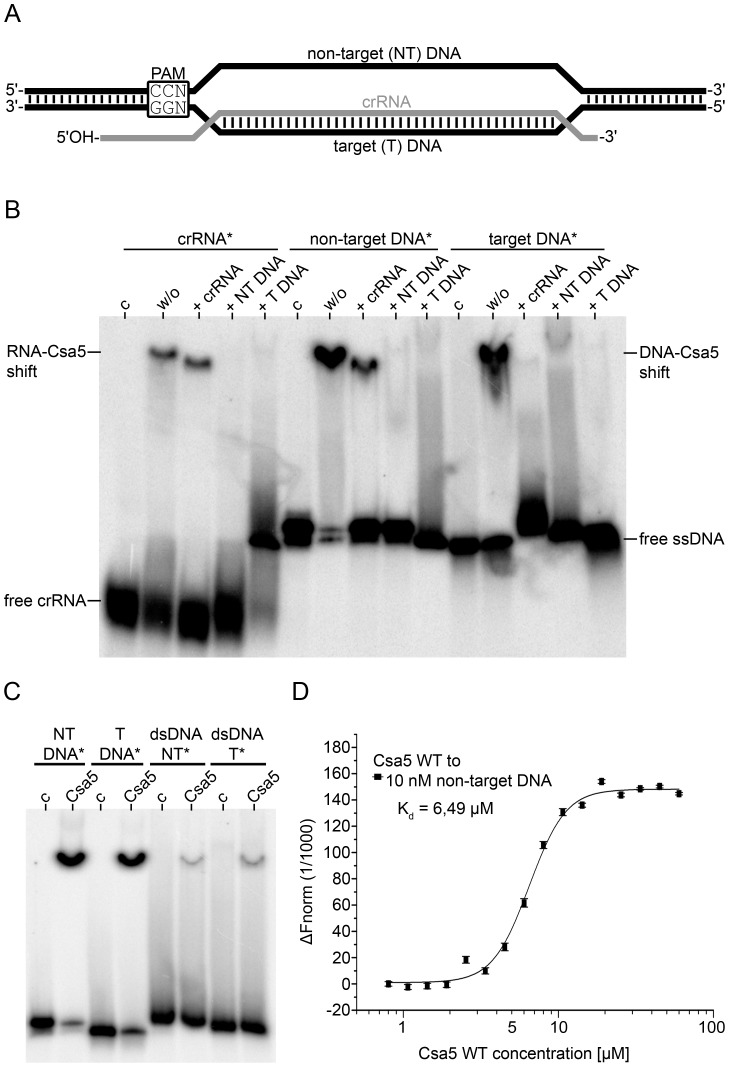
Nucleic acid binding analysis of Csa5. (A) Schematic illustration of an R-loop structure. The PAM sequence is located upstream of the protospacer sequence. (B) A competitive EMSA (6% native PAGE) was performed to study the affinity of Csa5 to different single-stranded nucleic acid molecules. Lanes (w/o) show labeled crRNA, non-target DNA (NT DNA) and target DNA (T DNA) incubated with 15 µM of Csa5 (37°C, 30 min) in absence of a competitor molecule. The shifted RNA and DNA signals represent the amount of RNA or DNA bound by the protein. Each binding event is further competed by a 1000-fold excess of unlabeled ssRNA (+ crRNA) or ssDNA (+ NT DNA/+ T DNA). Lanes (c) serve as a loading control. Asterisks indicate the labeled strand. (C) The binding affinity of the protein to ssDNA or dsDNA was investigated at a concentration of 15 µM Csa5 WT in an EMSA (6% native PAGE). (D) The dissociation constant K_d_ of the binding event of Csa5 to 10 nM of non-target DNA was determined by microscale thermophoresis. The graph shows the normalized fluorescent values of the non-target DNA at Csa5 concentrations from 0.8 to 60 µM obtained during MST analysis. The lower and upper baselines represent the unbound and bound state of the DNA, respectively. At the point of inflection 50% of the DNA is bound, revealing a K_d_ of 6.49 µM.

The Csa5 protein (TTX_1250) of *T. tenax* was biochemically characterized. Csa5 was heterologously produced in *E. coli*, and could be purified to apparent purity by heat precipitation (90°C) and two consecutive column chromatography steps (Fig. S2). Due to the observed interaction with nucleic acid and structural similarities of the *T. thermophilus* and *T. fusca* Cascade small subunit Cse2 to *S. solfataricus* Csa5 [31], [32], the nucleic acid binding properties of Csa5 were studied by electrophoretic mobility shift assays (EMSAs). The affinity to different nucleic acids that are components of an R-loop structure (crRNA and ssDNA) was tested (Fig. 2A; Table S2). The assays revealed that Csa5 binds to the crRNA and also binds to the two ssDNA fragments (non-target and target DNA) (Fig. 2B). The affinity for ssDNA substrates was higher than for crRNAs. This preference of Csa5 ssDNA binding was further analyzed in a competitive binding assay. While crRNA binding could not be outcompeted by a 1,000-fold excess of cold crRNA, it was possible to impede binding to crRNA with cold non-target DNA. In the opposite experiment, non-target DNA binding was outcompeted more efficiently by an excess of cold non-target DNA than an excess of cold crRNA. The competition of target DNA binding by the complementary crRNA or non-target DNA led to the formation of DNA:RNA or DNA:DNA duplexes, respectively. Csa5 binding to these double-stranded molecules was not observed. The affinity towards dsDNA was additionally tested in a separate assay by comparing the binding of Csa5 to the single-stranded non-target and target DNA strands to the hybridized double-stranded duplexes of both strands. This assay showed that the affinity of Csa5 to dsDNA is 14-fold weaker than its affinity to ssDNA (Fig. 2C). Next, the binding affinity of Csa5 to single-stranded DNA was measured via microscale thermophoresis, revealing a dissociation constant K_d_ of 6.5 µM (Fig. 2D), which matched the observed binding affinity in the EMSAs. Additionally, the effect of bivalent metal ions for supporting Csa5 binding was tested, but interaction with the non-target DNA strand was not found to be influenced by added metal ions (Fig. S3). One reason for the observed weak binding capacity of Csa5 to ssDNA *in vitro* might be that this isolated subunit requires the context of other Cascade subunits (e.g. the large subunit) for stabilization of the DNA interaction.

To further localize a potential Csa5 binding motif on the non-target strand, the 97 nt-long sequence was split into three shorter fragments of 38 nt in length (p1–p3). These fragments were bound by Csa5 with a comparable affinity, but 7-fold weaker in comparison to the longer non-target strand (Fig. S4). Additionally, a 19 nt-long fragment was tested (p4), which showed a similarly weak affinity (10-fold less) as fragments p1–p3. Thus, the binding of Csa5 to ssDNA seems to be rather sequence unspecific, but was found to have a higher affinity for longer substrates.

**Figure 3:**
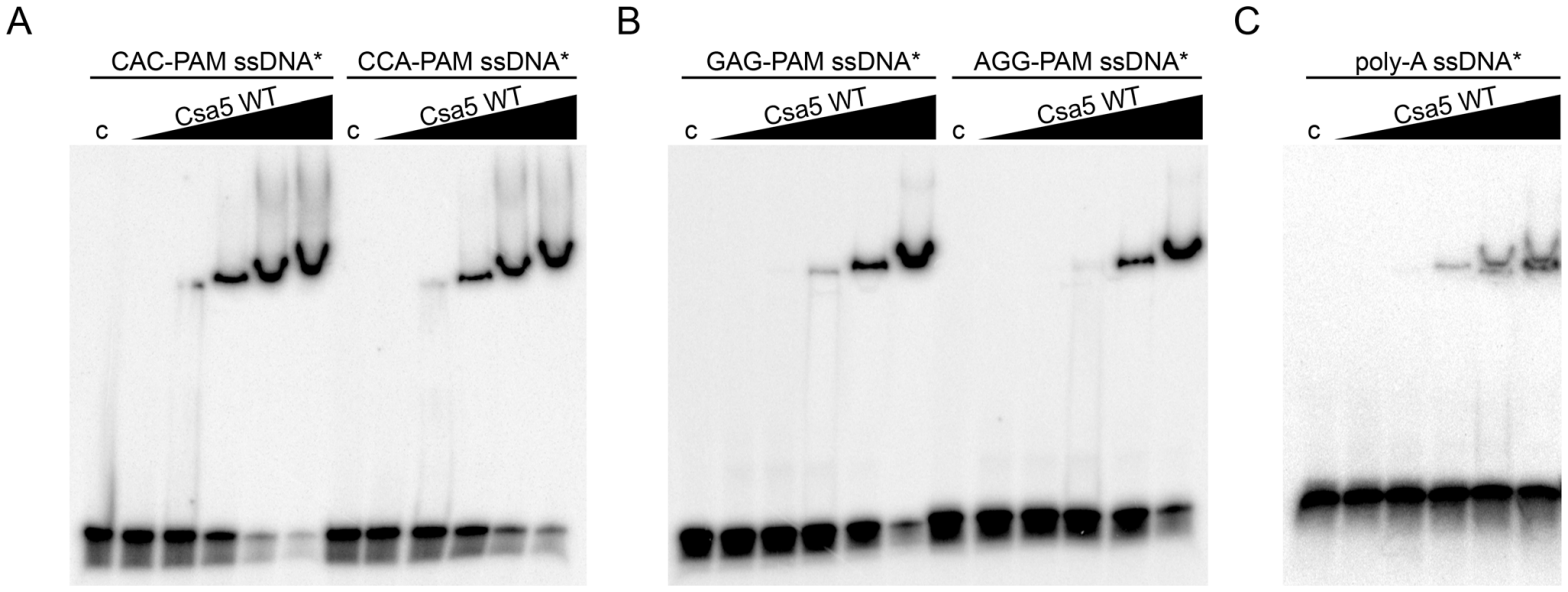
PAM binding analysis. The specificity of Csa5 for binding the PAM-motif was examined by using poly-PAM ((A) CCA-PAM (present on non-target DNA) and (B) AGG-PAM (present on target DNA)) ssDNA oligonucleotides in EMSAs (6% native PAGE). The disrupted poly-PAM (CAC-PAM and GAG-PAM) ssDNA oligonucleotides and a (C) poly-A ssDNA served as controls. Increasing concentrations (3.75, 7.5, 15, 30, 60 µM) of protein were tested. Asterisks indicate the labeled strand.

Based on the observed binding to ssDNA, we wanted to know if Csa5 has the ability to scan a DNA strand and recognize the PAM-motif. As we were able to define the functional PAM-motif for *in vitro* Cascade interference in *T. tenax*, consisting of a 5′-CCN-3′ sequence present on the non-target ssDNA strand upstream of the protospacer [25], the affinity for this motif was examined using an artificial poly-PAM ssDNA fragment comprising 16 PAM sequences (Table S2). Csa5 was found to bind this poly-PAM (5′-CCA-3′) as well as a disrupted PAM (5′-CAC-3′) substrate (Fig. 3A). Furthermore, poly-PAM substrates harboring the PAM (5′-AGG-3′) or disrupted PAM (5′-GAG-3′) on the target strand were tested, likewise showing no difference in the binding affinity of Csa5 (Fig. 3B). Binding was also observed for a poly-A ssDNA, underlining that the ssDNA binding activity of Csa5 appears to be sequence unspecific (Fig. 3C). Therefore, it seems unlikely that Csa5 is the elusive PAM-recognition protein of the *T. tenax* I-A Cascade, but it is feasible that this role rather requires interplay with other Cascade subunits.

**Figure 4:**
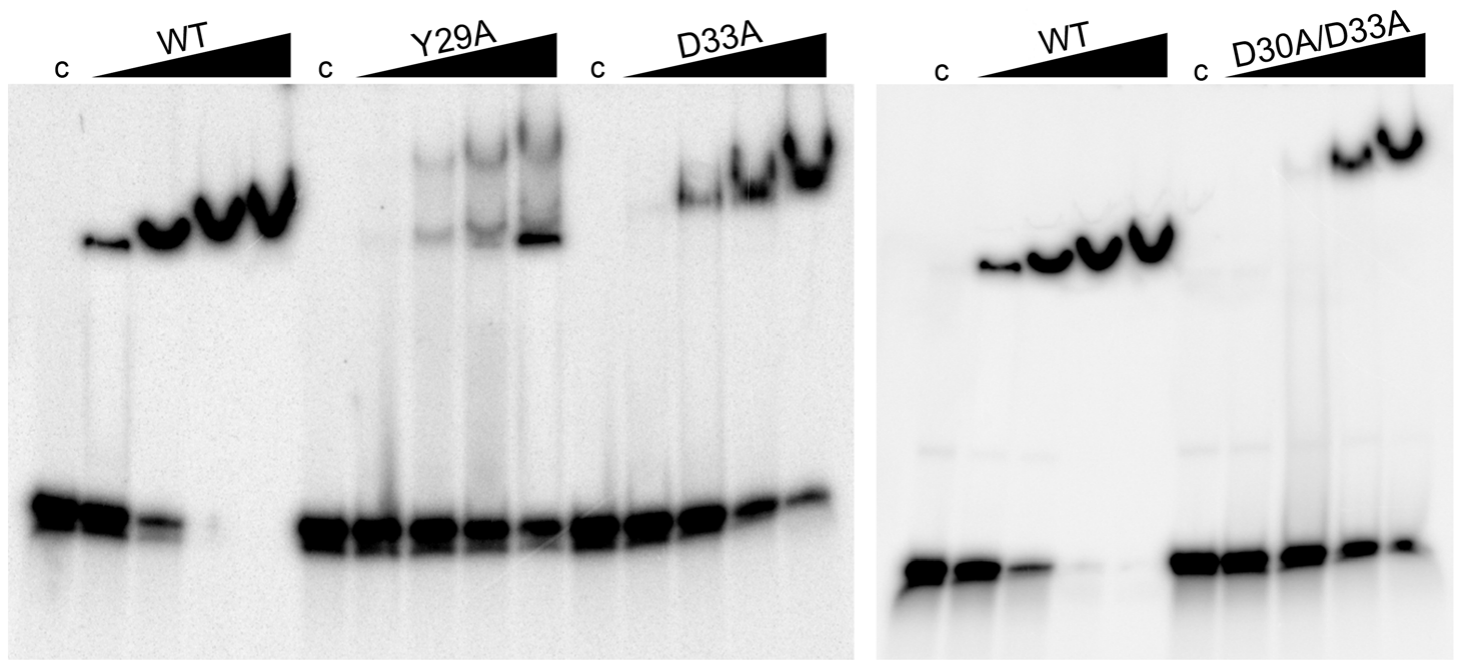
Impaired binding of Csa5 mutants. Concentrations from 7.5 to 60 µM of the purified Csa5 mutants Y29A, D33A and D30A/D33A were tested for binding to non-target DNA in EMSAs (6% native PAGE). A DNA-shift is observed for Csa5 WT from a concentration of 7.5 µM, while for Csa5 Y29A and D33A binding starts from 15 µM of protein. A defined shift can only be observed from a concentration of 30 µM for Csa5 D30A/D33A. At a concentration of 30 µM Csa5 WT the DNA substrate is fully bound, while the Csa5 mutants do not show complete binding at equal or higher protein concentrations.

Next, a mutational approach was applied to identify amino acids that are involved in nucleic acid binding. As potential candidates, we exchanged several conserved amino acids (Tyr29, Asp30, Asp33, Ala115) of the group I Csa5 sequences and one residue within a predicted coiled-coil structure (Leu94) of the protein (Fig. S1). All Csa5 mutants were produced, purified and tested in binding assays. The three mutants Y29A, D33A and D30A/D33A showed impaired binding to ssDNA (Fig. 4). In comparison to the Csa5 WT, the Y29A and D33A mutants only showed a binding affinity of around 10% and 18%, respectively. The strongest effect was observed for the D30A/D33A mutant, showing only about 4% binding compared to Csa5 WT. The EMSA analyses revealed a double shift for the Y29A mutant and reduced DNA migration for the D33A and D30A/D33A mutants (Fig. 4).

### Role of the small subunit Csa5 in Cascade

**Figure 5:**
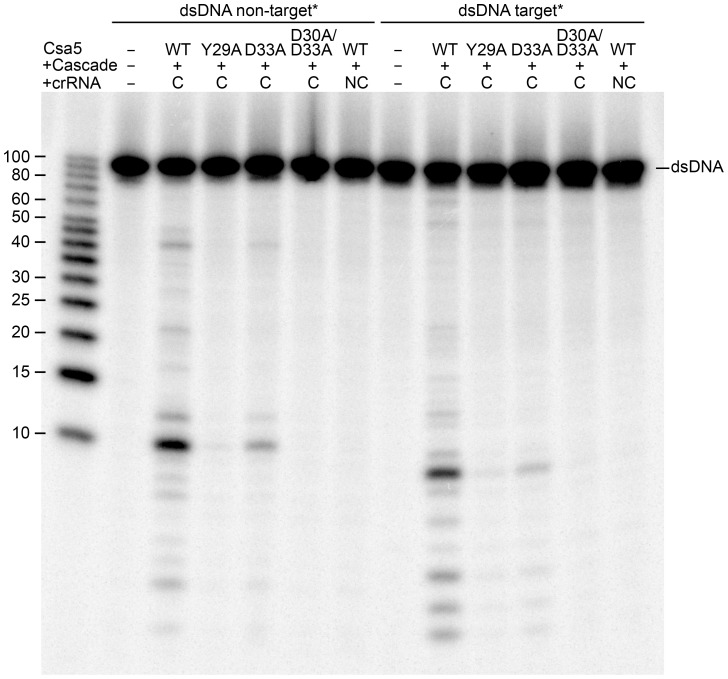
Interference assay with reconstituted Cascade complexes. The Csa5 constructed mutants (Y29A, D33A and D30A/D33A) were assembled into Cascade and tested for dsDNA cleavage. The assembled Cascade complex was loaded with complementary (C) crRNA 5.2 for 20 min at 80°C and the interference reaction was started by the addition of ATP, Mg^2+^, Mn^2+^ and the dsDNA substrate (int_5.2 CCT), which was either labeled on the non-target (forward) or the target strand (reverse) for 10 min at 70°C. The reaction products were separated on 20% denaturing gels. The non-complementary (NC) crRNA 5.13 was used as a control. Cleavage products for both strands are visible for Cascade containing Csa5 WT as reported previously [25]. A decreased cleavage activity can be observed for the Cascade complexes containing the Csa5 mutants. Asterisks indicate the labeled strand.

To verify that the observed ssDNA binding activity of Csa5 is functionally relevant within Cascade, the binding-impaired Csa5 mutants were tested in an *in vitro* interference assay for their effect on the degradation of protospacer DNA. Therefore, the purified Csa5 WT and the mutants Y29A, D33A and D30A/D33A were *in vitro* co-assembled with the missing Cascade subunits (Cas7, Cas5, Cas3′, Cas3″ and Cas8a2) to obtain the mature complex (Fig. S5). The amount of soluble assembled Cascade was similar for the wildtype and for the three Csa5 mutants, which indicates that complex formation was not impaired. The reconstituted complexes were then loaded with a mature crRNA (Table S2). Subsequently, the degradation of a crRNA-matching double-stranded protospacer DNA was monitored in a nuclease assay (Fig. 5). Interestingly, the degradation efficiency was significantly reduced for the Csa5 mutants. Cascade containing the Csa5 D33A mutant showed 6-fold less degradation efficiency, while a Cascade with incorporated Csa5 Y29A showed 18-fold less degradation of the dsDNA in comparison to the wildtype complex. The strongest effect was observed for Cascade containing the Csa5 D30A/D33A mutant, which showed nearly no cleavage of the dsDNA. Thus, ssDNA binding of Csa5 is required for efficient Cascade activity.

Based on these results, we hypothesize that *T. tenax* Csa5 fulfills a similar role in I-A Cascade as the small I-E subunit Cse2, which was proposed to interact with the R-loop [32]. Accordingly, Csa5 might stabilize the R-loop by binding to the displaced non-target DNA strand of the invading DNA.

### Recombinant Csa5 forms stable oligomers

Csa5 was found to form SDS-stable oligomers after several weeks of storage (Fig. S6A), and their identity was verified by mass spectrometry. The native molecular state of the protein was analyzed via size exclusion chromatography. Freshly purified Csa5 eluted at a retention volume of 16.8 ml, which corresponds to an estimated weight of 21 kDa, representing a monomer (theoretically 15.04 kDa) (Fig. S6B). The chromatogram of a three-month old sample showed two additional peaks at the retention volumes of 15.8 and 14.9 ml, corresponding to the size of a dimer (30 kDa) and trimer (42 kDa), respectively (Fig. S6B). Interestingly, oligomerization of Csa5 was also found in *S. solfataricus* [31]. Here, the crystal structure showed helical threads of Csa5 subunits that were formed by an intermonomeric salt bridge. Surprisingly, the two highly conserved amino acids D and R were involved in this salt bridge formation. This implied that oligomerization of Csa5 is a conserved feature, and initially led us to assume that the oligomerization of *T. tenax* Csa5 is also induced via salt bridge formation between these two amino acids. However, the produced Csa5 D33A mutant still exhibited oligomerization (Fig. S6B). Several attempts to deoligomerize the protein by biochemical approaches failed. The Csa5 oligomers were even present after treatment with 6 M GdmCl or after incubation at 120°C in SDS-buffer (Fig. S6C). An oligomerization of the protein by salt bridges is therefore unlikely. As GdmCl is one of the strongest reagents for protein unfolding, it appears that the oligomerization of Csa5 is probably not a conserved physiological feature, but rather is caused by the denaturation of the protein. This is supported by the observation that oligomerization of Csa5 can be induced by incubation at high temperatures (Fig. S6D). Usually, protein denaturing manifests as precipitation. However, precipitation could not be observed during the storage of the protein. It is possible that the protein structure collapses into a partially folded state during storage, described as molten globule conformation [37]. The collapsed structure might then lead to oligomerization of the protein. We tested oligomerized Csa5 for its capability to bind ssDNA. The protein was incubated with the non-target DNA strand at three temperatures (37, 70 and 90°C) for different time points (5, 30 and 60 min), which induced oligomerization of Csa5 visualized on the SDS-PAGE (Fig. S7A). The EMSA showed that only the monomeric protein efficiently bound the ssDNA (Fig. S7B). We therefore assume that the oligomerization of Csa5 is an artifact of the isolated protein, which might be prevented by the formation of the Cascade complex with the remaining Cas protein subunits, considering the high physiological temperature within *T. tenax*.

## Conclusions

We have identified Csa5 as a universal small subunit of I-A Cascade assemblies that is highly divergent at the sequence level. We could for the first time show that this protein binds nucleic acids with a preference for ssDNA, and that this binding activity is required during Cascade-mediated DNA decay. Csa5 oligomerization prevented ssDNA binding. We hypothesize that Csa5 utilizes its ssDNA binding activity in the Cascade context for R-loop stabilization, which would be of special importance in I-A CRISPR-Cas systems exclusively found in organisms living at elevated temperatures. It is possible that this is a general role of the small Cascade subunits that are also present in several other CRISPR-Cas subtypes.

## Supporting Information

Figure S1 **Multiple alignment of Csa5 sequences.** The three identified groups of archaeal Csa5 sequences are aligned and conserved residues are marked in blue. Listed are the genomic ORF numbers of the archaeal species. Amino acids that were altered for the generation of the *T. tenax* Csa5 mutants (TTX_1250) are marked with asterisks. With the exception of *Pyrobaculum sp. 1860* all Csa5 sequences share the conserved amino acids D and R.
(TIF)

Figure S2 **Purification of Csa5.** The SDS-PAGE shows the Csa5 WT and the mutants Csa5 Y29A, D33A, L94G, A115G and D30A/D33A after the last purification step via anion-exchange chromatography (MonoQ). The gel shows an apparent purity of all Csa5 variants.
(TIF)

Figure S3 **Effect of bivalent metal ions on Csa5 binding.** The binding of Csa5 to non-target DNA was investigated in the presence of 10 mM Ca^2+^, Mg^2+^, Zn^2+^, Mn^2+^ or Ni^2+^. The binding manner in the presence of the tested metals is comparable to the binding without metal ions (lane w/o). Asterisks indicate the labeled strand.
(TIF)

Figure S4 **Influence of the substrate size on Csa5 binding.** Substrates of different lengths were tested for Csa5 binding. The affinity to the longest substrate (non-target (NT) DNA; 97 nt) is significantly higher than to the truncated versions of this substrate (p1–p4). At a protein concentration of 15 µM about 85% of the non-target DNA is bound. In contrast, only 12% of the p1/2/3 DNA (38 nt) and 8% of the p4 DNA (19 nt) is bound at the same Csa5 concentration. Asterisks indicate the labeled strand.
(TIF)

Figure S5 **Reconstitution of Cascade complexes.** The picture shows the SDS-PAGE analysis of the reconstituted Cascade complexes containing Csa5 WT and the binding impaired mutants Csa5 Y29A, D33A and D30A/D33A.
(TIF)

Figure S6 **Oligomerization of Csa5.** (A) Purified Csa5 is analyzed via SDS-PAGE at different time points of storage (after 1, 2, 4, 8 and 16 weeks of storage at 4°C). SDS-stable dimers (30 kDa) are formed after the first week of storage. Trimer formation (45 kDa) becomes visible after eight weeks. (B) Gel filtration chromatograms of a freshly purified Csa5 WT solution (monomeric WT) and of three-month old Csa5 WT (oligomeric WT) and Csa5 D33A (oligomeric D33A) solutions. The freshly purified Csa5 WT solution shows a single peak at a retention volume of 16.8 ml. The chromatogram of the three-month old Csa5 WT and Csa5 D33A solutions show further peaks at around 15.8 and 14.9 ml. (C) Deoligomerization approach of a highly oligomerized Csa5 solution analyzed via SDS-PAGE. Depicted are biochemical attempts to deoligomerize a nine-month old protein solution (oligomeric) by GdmCl (6 M GdmCl), by additional incubation at 95°C (6 M GdmCl 95°C) and by 120°C incubation in an autoclave (120°C autocl.). A freshly purified (monomeric) Csa5 WT solution serves as control. The deoligomerization attempts fail, as the protein stays oligomeric. (D) SDS-PAGE analysis of freshly purified Csa5 WT and Csa5 D30A/D33A solutions incubated for 1 h at different temperatures (0°C, 37°C, 70°C, 90°C). For both protein purifications dimer and trimer formation is observed at 70°C and 90°C incubation, respectively, demonstrating that oligomerization of Csa5 can be induced by high temperature.
(TIF)

Figure S7 **Effect of Csa5 oligomerization on DNA binding.** Oligomerization of Csa5 was induced by incubation at high temperatures to study the effect of oligomerization on DNA binding. (A) SDS-PAGE of 15 µM Csa5 incubated with non-target DNA at different temperatures (37°C, 70°C and 90°C) and for different time points (5′, 30′, 60′). In the last four lanes the protein was pre-incubated for 1 h without DNA at the depicted temperatures, then DNA was added and incubated for 30 min at 37°C. Oligomerization of the protein can be observed for incubation at 70°C for 60 min and at 90°C for 30 and 60 min. Lane (c) shows the loading control without DNA. (B) EMSA (6% native PAGE) of identical reactions used in (A), but with labeled non-target DNA and lane (c) showing the loading control without protein. The DNA shifts show binding of Csa5 to DNA. Binding is lost for the reactions which show oligomerization of the protein in the SDS-PAGE.
(TIF)

Table S1 **Oligonucleotides for cloning of Csa5 mutants.**
(DOCX)

Table S2 **Oligonucleotides and RNA sequences for EMSA studies and** ***in vitro*** **interference assay.**
(DOCX)

Table S3 **Description of genome tags marked in the Csa5 phylogenetic tree (** **Figure 1** **).** Note: The identified Csa5 genes of other available *Sulfolobus islandicus* strains (*Csa5 duplicate of MI1425_0857: MI1627_0922; **Csa5 duplicate of MI1627_0966: LD85_0944, L215_0726, MI1425_0900, MI164_0909, YG5714_0762) were not listed in the phylogenetic tree due to nearly identical Csa5 sequences in the respective genomes.
(DOCX)
