# Supplementary figures and images for: DNA Binding Properties of the Small Cascade Subunit Csa5
Source: PLoS One. 2014 Aug 22;9(8):e105716. doi: 10.1371/journal.pone.0105716 (PMC4141822; doi:10.1371/journal.pone.0105716)

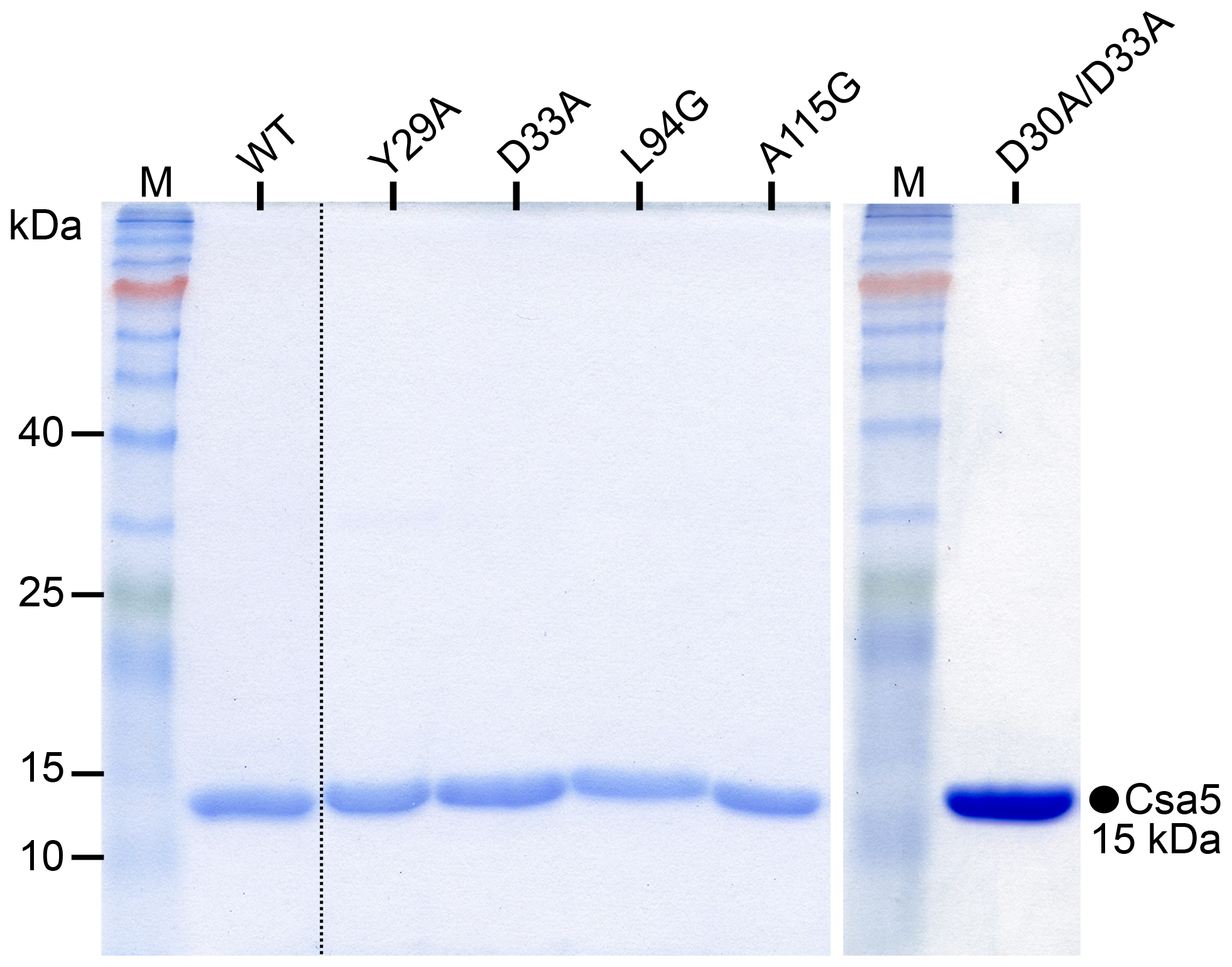

Supplement: Figure S2 — Purification of Csa5. The SDS-PAGE shows the Csa5 WT and the mutants Csa5 Y29A, D33A, L94G, A115G and D30A/D33A after the last purification step via anion-exchange chromatography (MonoQ). The gel shows an apparent purity of all Csa5 variants. (TIF) [file pone.0105716.s002.tif]

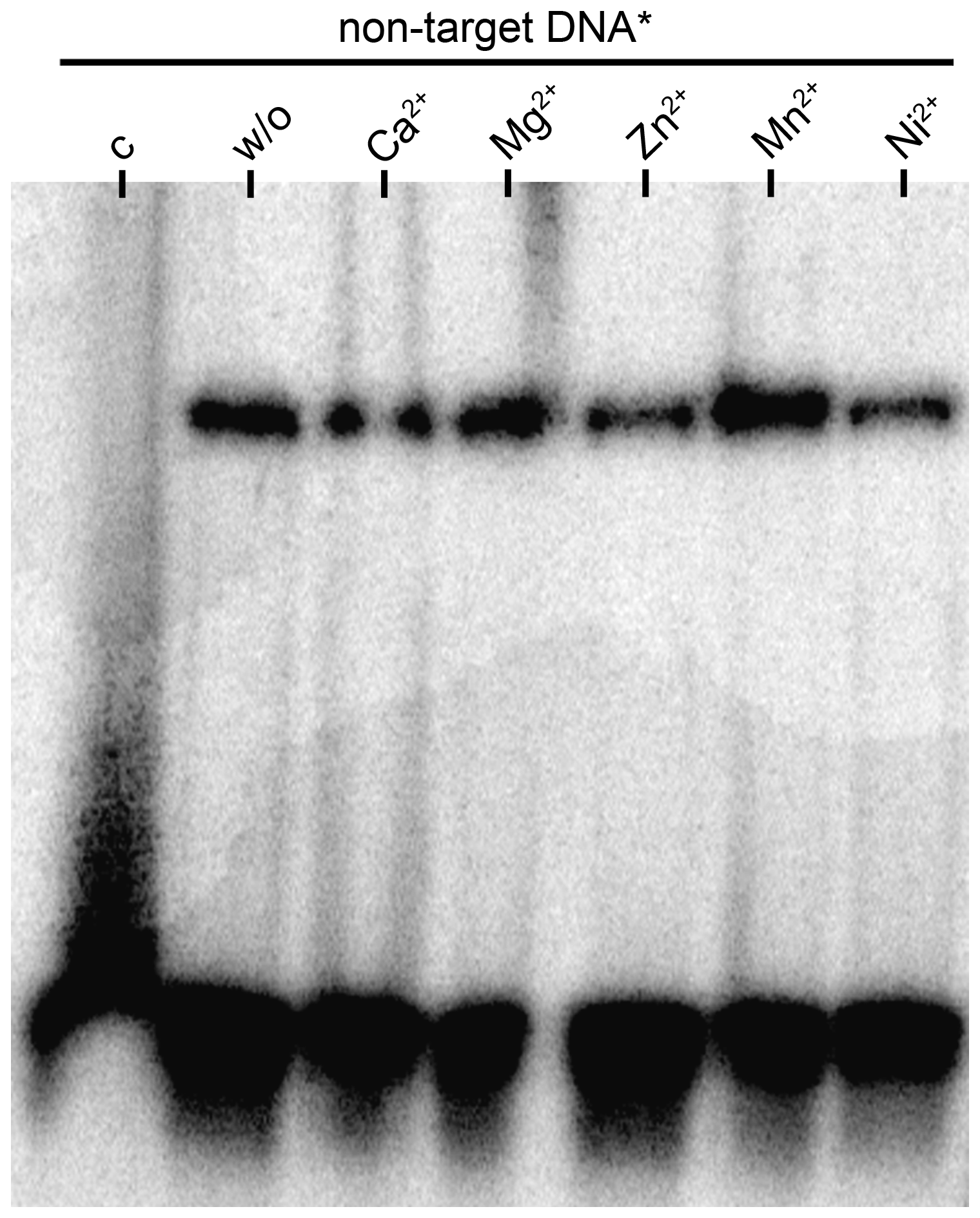

Supplement: Figure S3 — Effect of bivalent metal ions on Csa5 binding. The binding of Csa5 to non-target DNA was investigated in the presence of 10 mM Ca2+, Mg2+, Zn2+, Mn2+ or Ni2+. The binding manner in the presence of the tested metals is comparable to the binding without metal ions (lane w/o). Asterisks indicate the labeled strand. (TIF) [file pone.0105716.s003.tif]

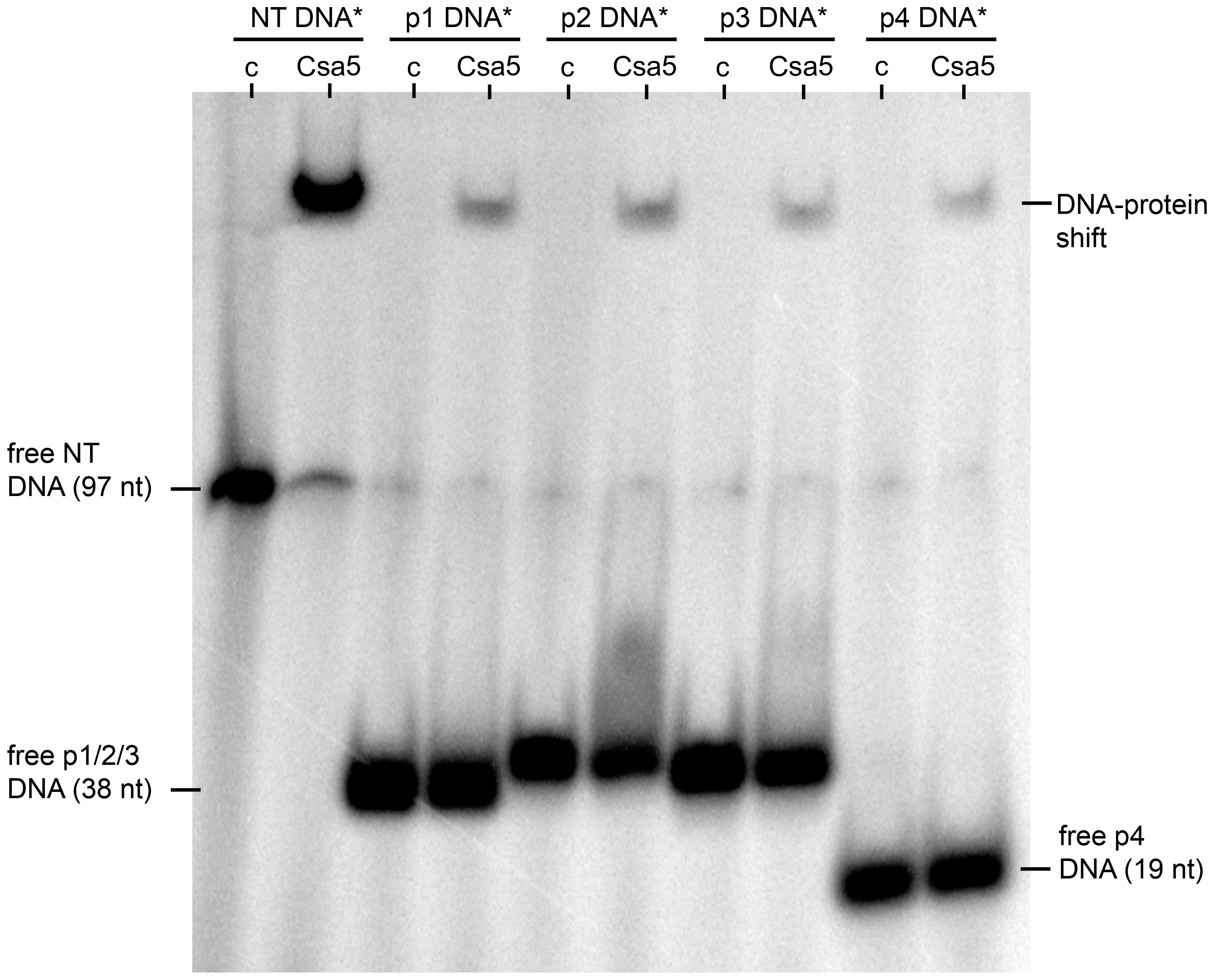

Supplement: Figure S4 — Influence of the substrate size on Csa5 binding. Substrates of different lengths were tested for Csa5 binding. The affinity to the longest substrate (non-target (NT) DNA; 97 nt) is significantly higher than to the truncated versions of this substrate (p1–p4). At a protein concentration of 15 µM about 85% of the non-target DNA is bound. In contrast, only 12% of the p1/2/3 DNA (38 nt) and 8% of the p4 DNA (19 nt) is bound at the same Csa5 concentration. Asterisks indicate the labeled strand. (TIF) [file pone.0105716.s004.tif]

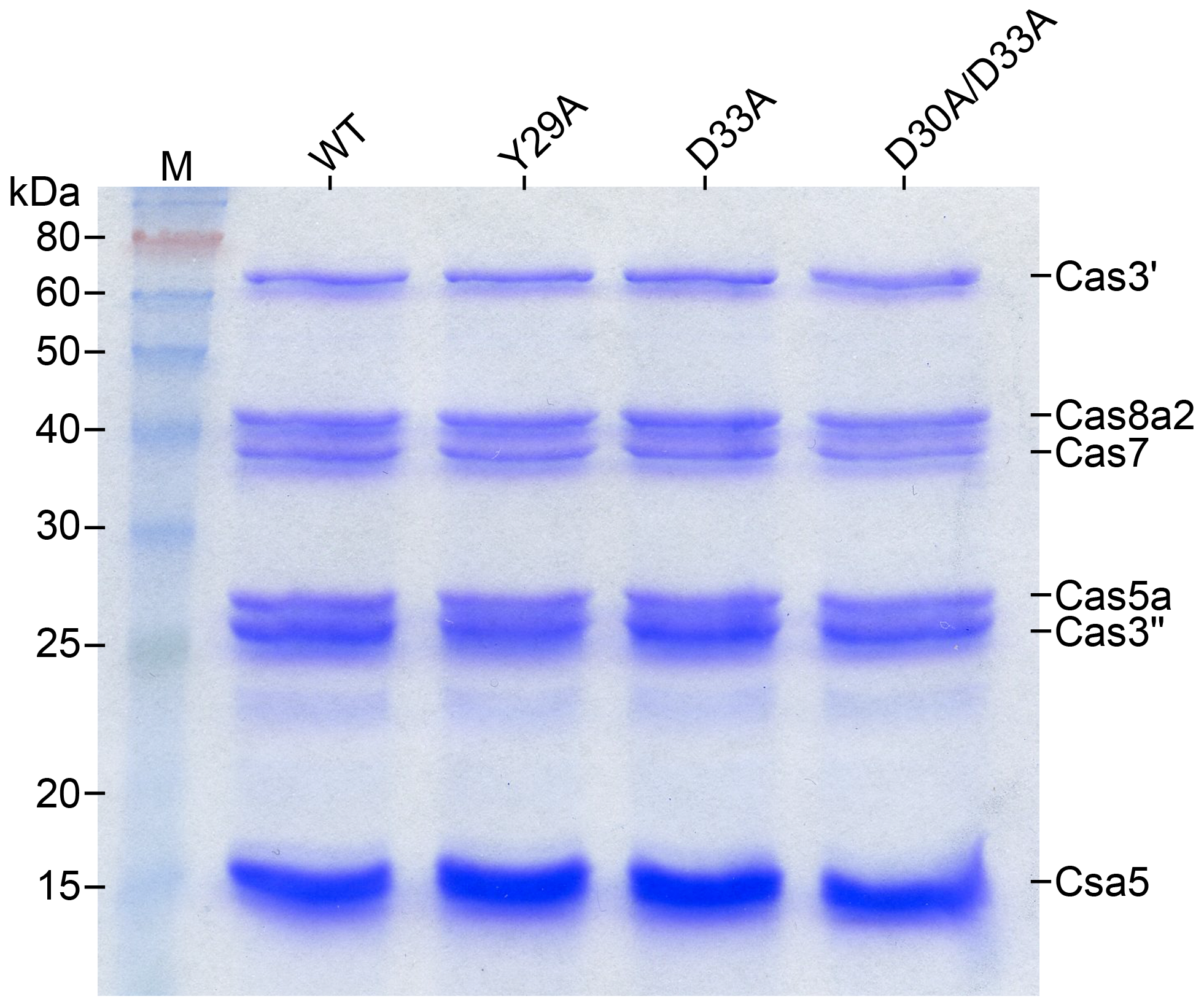

Supplement: Figure S5 — Reconstitution of Cascade complexes. The picture shows the SDS-PAGE analysis of the reconstituted Cascade complexes containing Csa5 WT and the binding impaired mutants Csa5 Y29A, D33A and D30A/D33A. (TIF) [file pone.0105716.s005.tif]

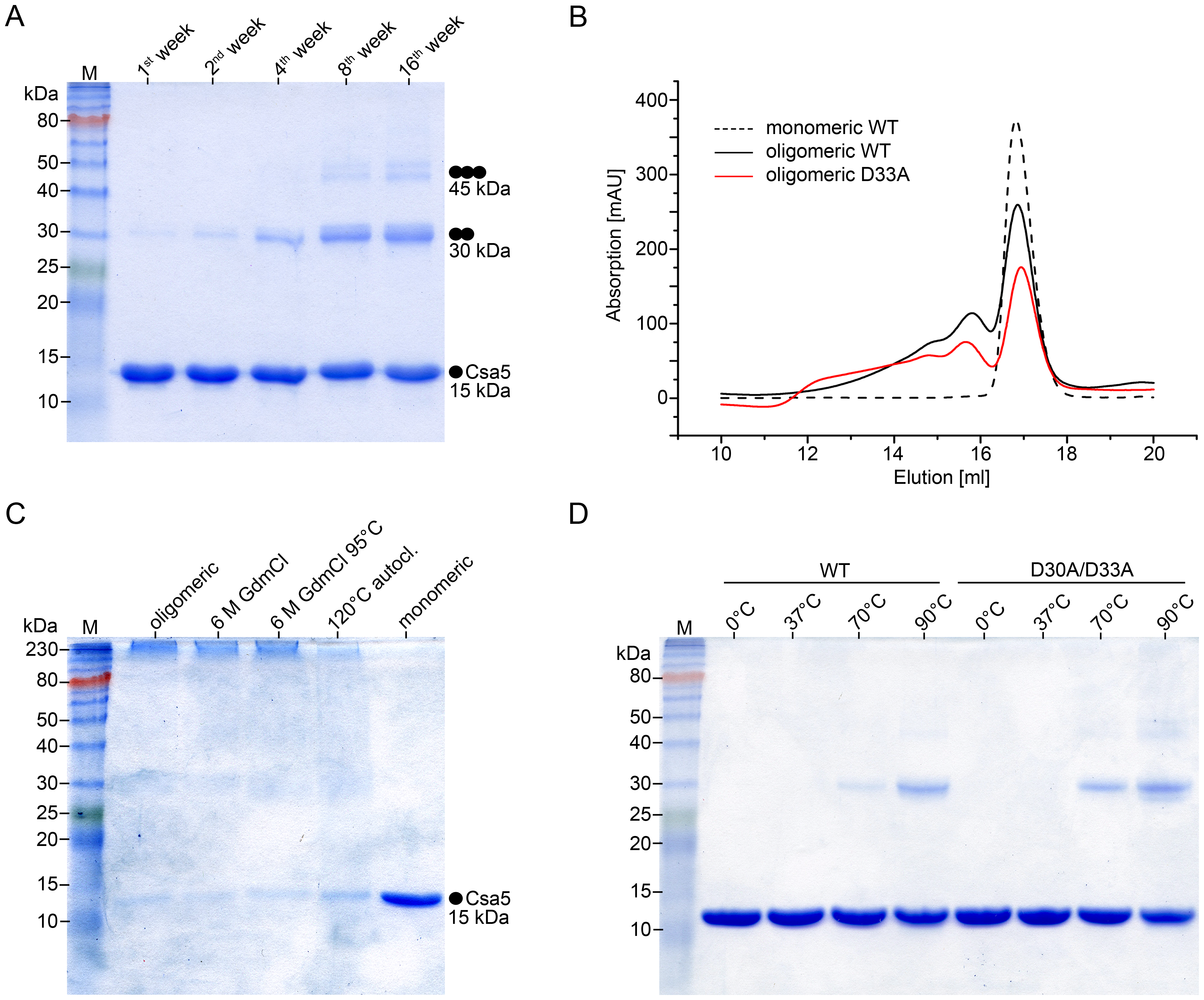

Supplement: Figure S6 — Oligomerization of Csa5. (A) Purified Csa5 is analyzed via SDS-PAGE at different time points of storage (after 1, 2, 4, 8 and 16 weeks of storage at 4°C). SDS-stable dimers (30 kDa) are formed after the first week of storage. Trimer formation (45 kDa) becomes visible after eight weeks. (B) Gel filtration chromatograms of a freshly purified Csa5 WT solution (monomeric WT) and of three-month old Csa5 WT (oligomeric WT) and Csa5 D33A (oligomeric D33A) solutions. The freshly purified Csa5 WT solution shows a single peak at a retention volume of 16.8 ml. The chromatogram of the three-month old Csa5 WT and Csa5 D33A solutions show further peaks at around 15.8 and 14.9 ml. (C) Deoligomerization approach of a highly oligomerized Csa5 solution analyzed via SDS-PAGE. Depicted are biochemical attempts to deoligomerize a nine-month old protein solution (oligomeric) by GdmCl (6 M GdmCl), by additional incubation at 95°C (6 M GdmCl 95°C) and by 120°C incubation in an autoclave (120°C autocl.). A freshly purified (monomeric) Csa5 WT solution serves as control. The deoligomerization attempts fail, as the protein stays oligomeric. (D) SDS-PAGE analysis of freshly purified Csa5 WT and Csa5 D30A/D33A solutions incubated for 1 h at different temperatures (0°C, 37°C, 70°C, 90°C). For both protein purifications dimer and trimer formation is observed at 70°C and 90°C incubation, respectively, demonstrating that oligomerization of Csa5 can be induced by high temperature. (TIF) [file pone.0105716.s006.tif]

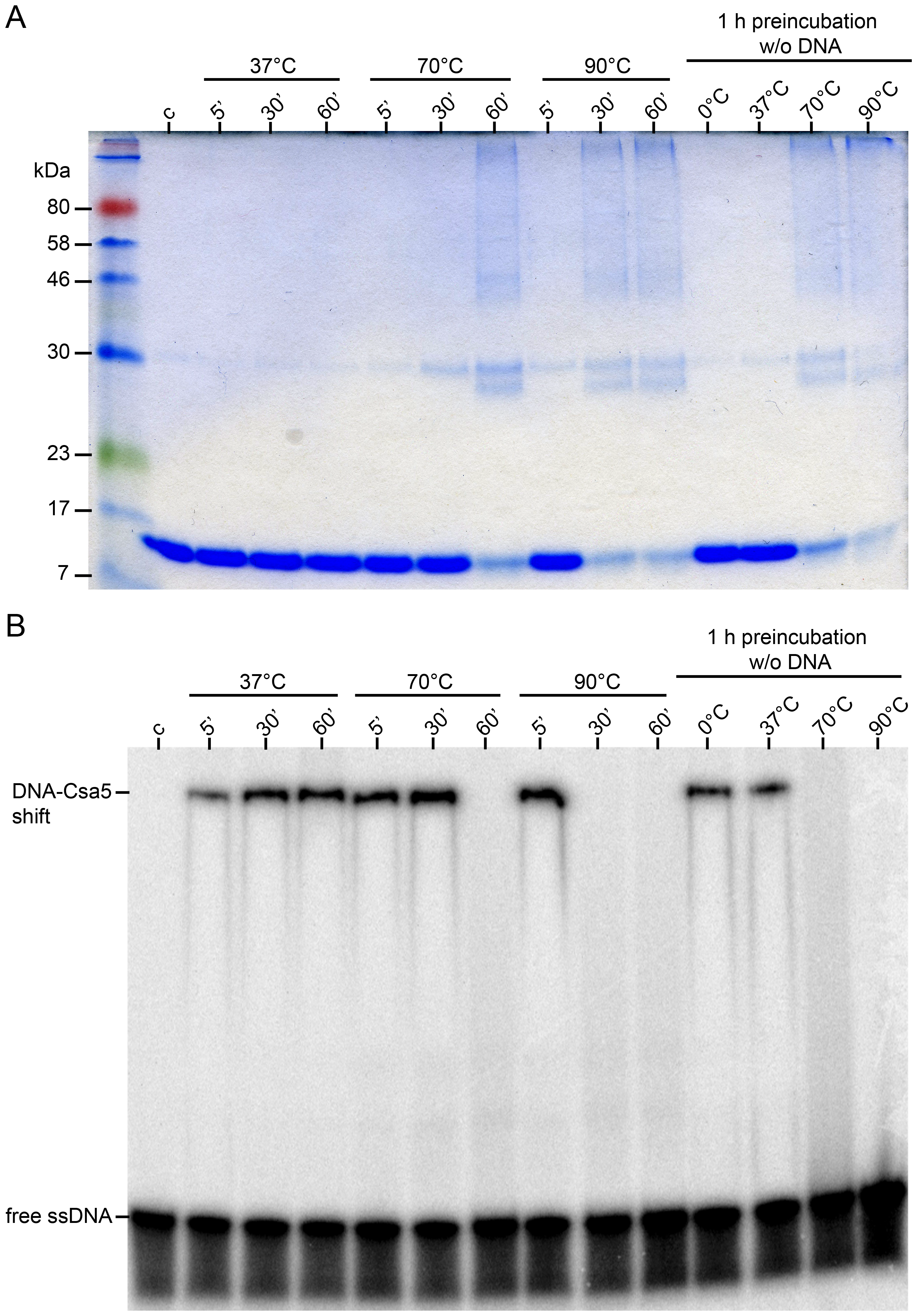

Supplement: Figure S7 — Effect of Csa5 oligomerization on DNA binding. Oligomerization of Csa5 was induced by incubation at high temperatures to study the effect of oligomerization on DNA binding. (A) SDS-PAGE of 15 µM Csa5 incubated with non-target DNA at different temperatures (37°C, 70°C and 90°C) and for different time points (5′, 30′, 60′). In the last four lanes the protein was pre-incubated for 1 h without DNA at the depicted temperatures, then DNA was added and incubated for 30 min at 37°C. Oligomerization of the protein can be observed for incubation at 70°C for 60 min and at 90°C for 30 and 60 min. Lane (c) shows the loading control without DNA. (B) EMSA (6% native PAGE) of identical reactions used in (A), but with labeled non-target DNA and lane (c) showing the loading control without protein. The DNA shifts show binding of Csa5 to DNA. Binding is lost for the reactions which show oligomerization of the protein in the SDS-PAGE. (TIF) [file pone.0105716.s007.tif]
