# Supplementary material for: DNA Binding Properties of the Small Cascade Subunit Csa5
Source: PLoS One. 2014 Aug 22;9(8):e105716. doi: 10.1371/journal.pone.0105716 (PMC4141822; doi:10.1371/journal.pone.0105716)
Supplement: Table S1 — Oligonucleotides for cloning of Csa5 mutants. (DOCX) [file pone.0105716.s008.docx]

**Table S1. Oligonucleotides for cloning of Csa5 mutants.**

| **Primer name** | **Sequence** |
| --- | --- |
| Csa5Y29A for | 5'-tacatctggagatggccgccgatgtgttggacgagc-3' |
| Csa5Y29A rev | 5'-gctcgtccaacacatcggcggccatctccagatgta-3' |
| Csa5D33A for | 5'-cctacgatgtgttggccgagctctccagatc-3' |
| Csa5D33A rev | 5'-gatctggagagctcggccaacacatcgtagg-3' |
| Csa5D30A/D33A for | 5'-ggagatggcctacgctgtgttggccgagc-3' |
| Csa5D30A/D33A rev | 5'-gctcggccaacacagcgtaggccatctcc-3' |
| Csa5L94G for | 5'-gctcgccaagcgcggtgaggaggcggat-3' |
| Csa5L94G rev | 5'-atccgcctcctcaccgcgcttggcgagc-3' |
| Csa5A115G for | 5'-gccatggccctaggtcccgacaaactg-3' |
| Csa5A115G rev | 5'-cagtttgtcgggacctagggccatggc-3' |
