# Supplementary material for: DNA Binding Properties of the Small Cascade Subunit Csa5
Source: PLoS One. 2014 Aug 22;9(8):e105716. doi: 10.1371/journal.pone.0105716 (PMC4141822; doi:10.1371/journal.pone.0105716)
Supplement: Table S2 — Oligonucleotides and RNA sequences for EMSA studies and in vitro interference assay. (DOCX) [file pone.0105716.s009.docx]

**Table S2. Oligonucleotides and RNA sequences for EMSA studies and *in vitro* interference assay.**

| **EMSA substrates** | **Sequence** |
| --- | --- |
| crRNA (5.13) (54 nt) | 5'OH−AUUGAAAGAUCGCUUGUCCAACCGGGCUCCUCUAUAUGUCGUCAUUAGCUUAGA−3' |
| non-target (NT) ssDNA (97 nt) | 5'−GTCGACTAATACGACTCACTATAGGCCTATCGCTTGTCCAACCGGGCTCCTCTATATGTCGTCATTAGCTTACCGCTGAGCAATAACTAGCAAGCTT−3' |
| target (T) ssDNA (97 nt) | 3'−CAGCTGATTATGCTGAGTGATATCCGGATAGCGAACAGGTTGGCCCGAGGAGATATACAGCAGTAATCGAATGGCGACTCGTTATTGATCGTTCGAA−5' |
| Cy3-non-target ssDNA (80 nt) | 5'Cy3−CGACTCACTATAGGCCTATCGCTTGTCCAACCGGGCTCCTCTATATGTCGTCATTAGCTTACCGCTGAGCAATAACTAGC−3' |
| p1 (part 1 of NT DNA) ssDNA (38 nt) | 5'−GTCGACTAATACGACTCACTATAGGCCTATCGCTTGTC−3' |
| p2 (part 2 of NT DNA) ssDNA (38 nt) | 5'−ATCGCTTGTCCAACCGGGCTCCTCTATATGTCGTCATT−3' |
| p3 (part 3 of NT DNA) ssDNA (38 nt) | 5'−CGTCATTAGCTTACCGCTGAGCAATAACTAGCAAGCTT−3' |
| p4 ssDNA (19 nt) | 5'−GGGGATCCTAATACGACTC−3' |
| CAC-PAM ssDNA  (100 nt) | 5'−AAAACACAAACACAAACACAAACACAAACACAAACACAAACACAAACACAAACACAAACACAAACACAAACACAAACACAAACACAAACACAAACACAAA−3' |
| CCA-PAM ssDNA  (100 nt) | 5'−AAAACCAAAACCAAAACCAAAACCAAAACCAAAACCAAAACCAAAACCAAAACCAAAACCAAAACCAAAACCAAAACCAAAACCAAAACCAAAACCAAAA−3' |
| GAG-PAM ssDNA  (100 nt) | 5'−AAAAGAGAAAGAGAAAGAGAAAGAGAAAGAGAAAGAGAAAGAGAAAGAGAAAGAGAAAGAGAAAGAGAAAGAGAAAGAGAAAGAGAAAGAGAAAGAGAAA−3' |
| AGG-PAM ssDNA  (100 nt) | 5'−AAAAGGAAAAGGAAAAGGAAAAGGAAAAGGAAAAGGAAAAGGAAAAGGAAAAGGAAAAGGAAAAGGAAAAGGAAAAGGAAAAGGAAAAGGAAAAGGAAAA−3' |
| **Interference assay substrates** | **Sequence** |
| crRNA (5.2) (50 nt) | 5'OH−AUUGAAAGCGUUGAUGCGGCCGCGACUGGCUGACUCAGCUAUUACGUUGA−3' |
| non-target (int_5.2 CCT for) ssDNA (93 nt) | 5'−GTCGACTAATACGACTCACTATAGACCTCGTTGATGCGGCCGCGACTGGCTGACTCAGCTATTACGTTCCGCTGAGCAATAACTAGCAAGCTT−3' |
| target (int_5.2 CCT rev) ssDNA (93 nt) | 3'−CAGCTGATTATGCTGAGTGATATCTGGAGCAACTACGCCGGCGCTGACCGACTGAGTCGATAATGCAAGGCGACTCGTTATTGATCGTTCGAA−5' |
