# Supplementary material for: DNA Binding Properties of the Small Cascade Subunit Csa5
Source: PLoS One. 2014 Aug 22;9(8):e105716. doi: 10.1371/journal.pone.0105716 (PMC4141822; doi:10.1371/journal.pone.0105716)
Supplement: Table S3 — Description of genome tags marked in the Csa5 phylogenetic tree ( Figure 1 ). Note: The identified Csa5 genes of other available Sulfolobus islandicus strains (*Csa5 duplicate of MI1425_0857: MI1627_0922; **Csa5 duplicate of MI1627_0966: LD85_0944, L215_0726, MI1425_0900, MI164_0909, YG5714_0762) were not listed in the phylogenetic tree due to nearly identical Csa5 sequences in the respective genomes. (DOCX) [file pone.0105716.s010.docx]

**Table S3. Description of genome tags marked in the Csa5 phylogenetic tree (Figure 1).**

| **genome tag** | **organism** | **ORF** |
| --- | --- | --- |
| **I.** |  |  |
| AF | *Archaeoglobus fulgidus DSM 4304* | AF1870 |
| APE | *Aeropyrum pernix K1* | APE_1236.1 |
| Kcr | *Candidatus Korarchaeum cryptofilum OPF8* | Kcr_0428 |
| PAE | *Pyrobaculum aerophilum str. IM2* | PAE0211 |
| Pogu | *Pyrobaculum oguniense TE7* | Pogu_1148 |
| Smar | *Staphylothermus marinus F1* | Smar_0320 |
| Tneu_b | *Pyrobaculum neutrophilum V24Sta* | Tneu_1137 |
| TTX_b | *Thermoproteus tenax Kra 1* | TTX_1250 |
| Vdis | *Vulcanisaeta distributa DSM 14429* | Vdis_1694 |
| **II.** |  |  |
| Cmaq | *Caldivirga maquilingensis IC-167* | Cmaq_1521 |
| Desfe | *Desulfurococcus fermentans DSM 16532* | Desfe_1017 |
| Desmu | *Desulfurococcus mucosus DSM 2162* | Desmu_0993 |
| DKAM | *Desulfurococcus kamchatkensis 1221n* | DKAM_0778 |
| Ferp | *Ferroglobus placidus DSM 10642* | Ferp_1867 |
| Igag | *Ignisphaera aggregans DSM 17230* | Igag_0913 |
| MJ | *Methanocaldococcus jannaschii DSM 2661* | MJ_0380 |
| P186 | *Pyrobaculum sp. 1860* | P186_0959 |
| Pars | *Pyrobaculum arsenaticum DSM 13514* | Pars_1126 |
| Pcal | *Pyrobaculum calidifontis JCM 11548* | Pcal_1272 |
| PF | *Pyrococcus furiosus DSM 3638* | PF0643 |
| PNA2 | *Pyrococcus sp. NA2* | PNA2_1821 |
| Tagg | *Thermosphaera aggregans DSM 11486* | Tagg_0804 |
| TCELL | *Thermogladius cellulolyticus 1633* | TCELL_1019 |
| TGAM | *Thermococcus gammatolerans EJ3* | TGAM_1291 |
| Tneu_a | *Pyrobaculum neutrophilum V24Sta* | Tneu_0993 |
| Tpen | *Thermofilum pendens Hrk 5* | Tpen_1357 |
| TTX_a | *Thermoproteus tenax Kra 1* | TTX_0236 |
| VMUT | *Vulcanisaeta moutnovskia 768-28* | VMUT_1396 |
| **III.** |  |  |
| Ahos | *Acidianus hospitalis W1* | Ahos_1746 |
| Hbut | *Hyperthermus butylicus DSM 5456* | Hbut_0645 |
| Igni | *Ignicoccus hospitalis KIN4/I* | Igni_1141 |
| Mcup | *Metallosphaera cuprina Ar-4* | Mcup_1150 |
| Mefer | *Methanocaldococcus fervens AG86* | Mefer_1437 |
| MFS | *Methanocaldococcus sp. FS406-22* | MFS40622_1173 |
| Msed | *Metallosphaera sedula DSM 5348* | Msed_1144 |
| PYCH | *Pyrococcus yayanosii CH1* | PYCH_09610 |
| Pyrfu_a | *Pyrolobus fumarii 1A* | Pyrfu_0429 |
| Pyrfu_b | *Pyrolobus fumarii 1A* | Pyrfu_0510 |
| SI_M1425 | *Sulfolobus islandicus M.14.25* | MI1425_0857* |
| SI_M1627 | *Sulfolobus islandicus M.16.27* | MI1627_0966** |
| SI_YN1551 | *Sulfolobus islandicus Y.N.15.51* | YN1551_2116 |
| SSO_a | *Sulfolobus solfataricus P2* | SSO_1398 |
| SSO_b | *Sulfolobus solfataricus P2* | SSO_1443 |
| SSO_c | *Sulfolobus solfataricus P2* | SSO_1996 |
| ST_a | *Sulfolobus tokodaii str. 7* | ST_0028 |
| ST_b | *Sulfolobus tokodaii str. 7* | ST_2636 |
| TK | *Thermococcus kodakarensis KOD1* | TK0454 |
